# Laser therapy for genitourinary syndrome of menopause: systematic review and meta-analysis of randomized controlled trial

**DOI:** 10.61622/rbgo/2024rbgo38

**Published:** 2024-05-27

**Authors:** Lisieux de Lourdes Martins Nóbrega Pessoa, Amaxsell Thiago Barros de Souza, Ayane Cristine Alves Sarmento, Ana Paula Ferreira Costa, Isis Kelly dos Santos, Eduardo Pereira de Azevedo, Kleyton Santos de Medeiros, Ana Katherine Gonçalves, Ricardo Ney Cobucci

**Affiliations:** 1 Universidade Federal do Rio Grande do Norte Natal RN Brazil Universidade Federal do Rio Grande do Norte, Natal, RN, Brazil.; 2 Universidade Potiguar Natal RN Brazil Universidade Potiguar, Natal, RN, Brazil.; 3 Liga Norteriograndense contra o Câncer Natal RN Brazil Liga Norteriograndense contra o Câncer, Natal, RN, Brazil.

**Keywords:** Menopause, Laser therapy, Postemenopause, Female urogenital diseases

## Abstract

**Objective:**

This meta-analysis of randomized controlled trials (RCTs) aimed to update evidence on the effectiveness and safety of laser therapy for treating genitourinary syndrome of menopause (GSM).

**Data sources:**

Manuscripts published until May 2023 were systematically searched in PubMed; Embase; Scopus; Web of Science; CENTRAL; CINAHL; and clinical trial databases (www.trialscentral.org, www.controlled-trials.com, and clinicaltrials.gov), with no language and year of publication restriction.

**Studies selection:**

RCTs with women diagnosed with GSM, and the intervention was vaginal laser therapy (CO2-laser or Er: YAG-laser) comparing with placebo (sham therapy), no treatment or vaginal estrogen therapy.

**Data collection:**

Two authors evaluated the publications for inclusion based on the title and abstract, followed by reviewing the relevant full-text articles. Disagreements during the review process were addressed by consensus, with the involvement of a third author.

**Data synthesis:**

Twelve RCTs, representing a total of 5147 participants, were included in this review. Vaginal health index (VHI) significantly improved in the carbon dioxide laser (CO2-laser) therapy group (MD=2.21; 95% CI=1.25 to 3.16), while dyspareunia (MD=−0.85; 95% CI=−1.59 to −0.10), dryness (MD=−0.62; 95% CI=−1.12 to −0.12) and burning (MD= −0.64; 95% CI=−1.28 to −0.01) decreased. No serious adverse effects were reported.

**Conclusion:**

CO2-laser increases VHI score and decreases dyspareunia, dryness and burning, especially when compared to sham-laser. However, the certainty of the evidence is low, thus preventing the recommendation of laser therapy for GSM management.

## Introduction

The genitourinary syndrome of menopause (GSM) is a condition that affects about 50% of postmenopausal women because of hypoestrogenism in the tissues, which reduces elastin and collagen, resulting in thinner vaginal epithelium and higher vaginal pH.^(1,2)^ The main vulvovaginal symptoms include vaginal pruritus, dyspareunia, dryness, itching, urinary incontinence, and recurrent urinary tract infections.^(2)^ The severity of vulvovaginal symptoms is likely to increase over time and such symptoms have been associated with poor quality of life and mental health problems in postmenopausal women.^(2,3)^

The available treatment for GSM includes non-hormonal therapies (vaginal lubricants, moisturizers and ospemifene), as well as different hormone therapies.^(4,5)^ The evidence regarding the long-term effects of vaginal estrogen use on endometrial safety is limited, and the adherence rate varies from 52% to 74%.^(2,6)^ In addition, some women refuse to undergo hormone therapy or are at a high risk of complications.^(2)^

Vaginal laser therapy is a treatment that has been used to reduce symptoms of GSM. Its mechanism of action involves the creation of a microtrauma that induces the thickening of epithelium, blood vessel formation, and collagen synthesis, which stimulate the body's mechanisms of tissue repair, growth, and healing.^(2,7,8)^ The two types of lasers that have been mostly evaluated for GSM treatment are carbon dioxide laser (CO2-laser) and the Erbium: YAG (Er: YAG) laser.^(9)^ Due to the current scarcity of available evidence, vaginal laser therapies are not recommended for treating the symptoms of GSM by the North American Menopause Society (NAMS) and are not approved by the US Food and Drug Administration (FDA).^(2)^

Four recently published systematic reviews had omitted relevant studies and also presented some flaws in the methodology. The first systematic review included 12 prospective studies with a total of 459 participants, the second one included six randomized clinical trials (RCTs) and a total of 270 women with GSM, the third with 10 controlled intervention studies, 7 observational cohort and cross-sectional studies and 47 before-after studies without a control group, whereas the fourth systematic review included only 3 RCT. These systematic reviews suggested that vaginal laser treatment may be effective for postmenopausal women with GSM signs and symptoms.^(10–13)^ However, in the reviews performed only with RCT, the authors concluded that further randomized trials with larger sample sizes are required to investigate whether vaginal laser therapy could be a potential treatment alternative for women with contraindications to vaginal estrogen treatment and other hormonal therapies.^(11,13)^

Another systematic review published in 2022, which included only 4 studies with no RCTs, investigated the effect of vaginal CO2-laser on the management of GSM in gynecological cancer patients. The authors concluded that there is a lack of enough evidence in the literature to support the impact and safety of the use of vaginal CO2-laser in this population.^(14)^ Therefore, there is a clear need to update the evidence that would ultimately guide health professionals on the efficacy and safety of vaginal laser in the treatment of GSM.

Thus, this RCTs systematic review aimed to update the evidence on the use of vaginal laser to relieve the signs and symptoms of GSM by including studies that were left out of the previous articles and, therefore, to verify the certainty of this evidence for the main outcomes involved in such therapy.

## Methods

This systematic review and meta-analysis of randomized controlled trials (RCT) was conducted in accordance with the recommendations of the Cochrane Handbook for Systematic Reviews of Interventions^(15)^ and 2020 Preferred Reporting Items for Systematic Review and Meta-analysis (PRISMA) statement.^(16)^ The protocol was prospectively registered through the International Prospective Register of Systematic Reviews (PROSPERO/CRD42021253605) and was previously published in a scientific journal.^(17)^

The review question was: "Is laser therapy an effective and safe option for treating GSM?" The question was formulated based on the PICOS framework, and the elements were as follow:

- Population/participants: women with GSM;
- Intervention: laser therapy (CO2-laser/ Er: YAG).
- Comparison: no treatment, placebo, vaginal estrogen therapy (VET);
- Outcome: vaginal pH, vaginal atrophy, dryness, dyspareunia, itching, burning, female sexual function index (FSFI), dysuria, urinary frequency, urinary urgency, urinary incontinence, urinary tract infections, adverse events, and drop-outs due to adverse events;
- Study design: randomized clinical trials.

### Studies selection

Randomized controlled trials with women diagnosed with GSM, according to new terminology for vulvovaginal atrophy from the International Society for the Study of Women's Sexual Health and The North American Menopause Society.^(18)^ The trials had to use vaginal laser therapy (CO2-laser or Er: YAG-laser) comparing with placebo (sham-laser), no treatment or vaginal estrogen therapy. Narrative and systematic reviews, conference abstracts, brief communications, ongoing and preprint RCT or manuscripts with incomplete data and insufficient information were excluded.

Vaginal atrophy was considered the primary outcome, being evaluated in the RCTs through the vaginal health index questionnaire (VHI), which consists of five measurements: elasticity, fluid volume, pH, epithelial integrity, and moisture. The following were considered as secondary outcomes:

- Urinary incontinence, as determined using micturition diaries, the Urinary Distress Inventory-6 (UDI-6), and the International Consultation on Incontinence Questionnaire Urinary Incontinence Short Form (ICIQ-UI SF);
- Dyspareunia and dryness, which were evaluated using three different visual analog scale (VAS): 0-10, 0-5 and 0-3;
- Itching, burning, and dysuria, which were evaluated using the 0-10 Vaginal Assessment Scale (VAS);
- Female sexual function index (FSFI), that measure sexual functioning of women in six different domains: desire, arousal, lubrication, orgasm, satisfaction and pain;^(19)^
- Frequency and urinary urgency, evaluated in RCTs with different methodologies, such as micturition diaries, the Overactive Bladder Questionnaire Short Form (OAB-Q SF), the ICIQ- Female Lower Urinary Tract Symptoms (ICIQ-FLUTS),^(20)^ and the UDI-6;
- Urinary tract infections, assessed through urine culture;
- Adverse events and drop-outs due to side effects.

### Data sources

The manuscripts published from inception until May 2023 were systematically searched in the following databases: PubMed; Embase; Scopus; Web of Science; the Cochrane Central Register of Controlled Trials (CENTRAL); CINAHL; and clinical trial databases (www.trialscentral.org, www.controlled-trials.com, and clinicaltrials.gov). Reference lists of relevant primary studies and review articles were searched manually with no restriction regarding the language and year of publication. The search was performed with a combination of Medical Subject Headings (MESH) and "entry terms". The complete electronic search strategy for each database is presented in (Chart 1).

**Chart 1:** Search strategies

	Cochrane Library search strategy
**Number**	**Search items**
1	Postmenopausal
2	Postmenopausal women
3	Menopausal genitourinary syndrome
4	Vaginal atrophy
5	Vulvovaginal atrophy
6	OR/1-6
7	Laser
8	Laser therapy
9	Vaginal laser therapy
10	OR/7-10
11	pH
12	Dyspareunia
13	Itching
14	Burning
15	Dysuria
16	Urinary tract infections
17	Urinary frequency
18	Urinary incontinence
19	Vulvovaginal atrophy
20	6 AND 10 AND 20
	**EMBASE search strategy**
**Number**	**Search items**
1	Postmenopausal
2	Postmenopausal women
3	Menopausal genitourinary syndrome
4	Vaginal atrophy
5	Vulvovaginal atrophy
6	OR/1-6
7	Laser
8	Laser therapy
9	Vaginal laser therapy
10	OR/7-10
11	pH
12	Dyspareunia
13	Itching
14	Burning
15	Dysuria
16	Urinary tract infections
17	Urinary frequency
18	Urinary incontinence
19	Vulvovaginal atrophy
20	6 AND 10 AND 20
	**MEDLINE search strategy**
**Number**	**Search items**
1	Postmenopausal
2	Postmenopausal women
3	Menopausal genitourinary syndrome
4	Vaginal atrophy
5	Vulvovaginal atrophy
6	OR/1-6
7	Laser
8	Laser therapy
9	Vaginal laser therapy
10	OR/7-10
11	pH
12	Dyspareunia
13	Itching
14	Burning
15	Dysuria
16	Urinary tract infections
17	Urinary frequency
18	Urinary incontinence
	**MEDLINE search strategy**
**Number**	**Search items**
19	Vulvovaginal atrophy
20	6 AND 10 AND 20
	**Scopus search strategy**
**Number**	**Search items**
1	Postmenopausal
2	Postmenopausal women
3	Menopausal genitourinary syndrome
4	Vaginal atrophy
5	Vulvovaginal atrophy
6	OR/1-6
7	Laser
8	Laser therapy
9	Vaginal laser therapy
10	OR/7-10
11	pH
12	Dyspareunia
13	Itching
14	Burning
15	Dysuria
16	Urinary tract infections
17	Urinary frequency
18	Urinary incontinence
19	Vulvovaginal atrophy
20	6 AND 10 AND 20
	**Web of Science search strategy**
**Number**	**Search items**
1	Postmenopausal
2	Postmenopausal women
3	Menopausal genitourinary syndrome
4	Vaginal atrophy
5	Vulvovaginal atrophy
6	OR/1-6
7	Laser
8	Laser therapy
9	Vaginal laser therapy
10	OR/7-10
11	pH
12	Dyspareunia
13	Itching
14	Burning
15	Dysuria
16	Urinary tract infections
17	Urinary frequency
18	Urinary incontinence
19	Vulvovaginal atrophy
20	6 AND 10 AND 20
	**Clinical trial databases search strategy**
**Number**	**Search items**
1	Postmenopausal
2	Postmenopausal women
3	Menopausal genitourinary syndrome
4	Vaginal atrophy
5	Vulvovaginal atrophy
6	OR/1-6
7	Laser
8	Laser therapy
9	Vaginal laser therapy
10	OR/7-10
11	6 AND 10

### Data collection

The retrieved literature was imported into Rayyan (https://www.rayyan.ai/) software, from which the duplicate articles were eliminated. Two authors evaluated the publications for inclusion based on the title and abstract, followed by reviewing the relevant full-text articles. In addition, the two authors manually searched the references of each article for potential other eligible studies. Disagreements during the review process were addressed by consensus, with the involvement of a third author.

Two authors extracted data independently, and the extracted results were checked by a third author. A standardized data extraction form was used to collect the following data: names of authors; year of publication; country; study design; sample, mean age (in years); inclusion criteria; therapeutic protocol; follow-up; and outcomes. In case of additional information was needed, the corresponding author was contacted by email. Three authors independently assessed the risk of bias of the selected studies using the Cochrane risk of bias tool.^(21)^ Disagreements were resolved by consensus, with the involvement of a fourth author. Certainty of evidence was graded using the Grading of Recommendations, Assessment, Development and Evaluation (GRADE) approach for primary outcomes and serious adverse events.^(22)^

For each included RCTs, continuous outcomes were presented as mean ± standard deviation, mean differences (MD), standardised mean diferences (SMD), or hazard ratios (HR) with inverse-variance fixed-effect or random-effects analysis and dichotomous outcomes as risk ratios (RRs) with Mantel-Haenszel random-effects analysis and 95% confidence intervals (CI) for all outcome measurements. The heterogeneity among studies was quantified using Cochran's Q test and the inconsistency I2 test. When I2 was between 0 and 50%, the heterogeneity was acceptable. The funnel plots, used when more than 10 studies were included in the meta-analysis, were adopted to assess the publication bias. For data with an asymmetric funnel plot, Egger's linear regression test was additionally performed. The trim and fill method was used to correct publication bias. Sensitivity analyses were carried out to find out whether the quality of each eligible study might influence the result. A sensitivity analysis was conducted removing each individual RCTs to ensure that a single study did not affect the overall meta-analysis. Statistical analyses were performed using Review Manager (RevMan) software version 5.4 and STATA version 16.1.

## Results

**Figure 1:**
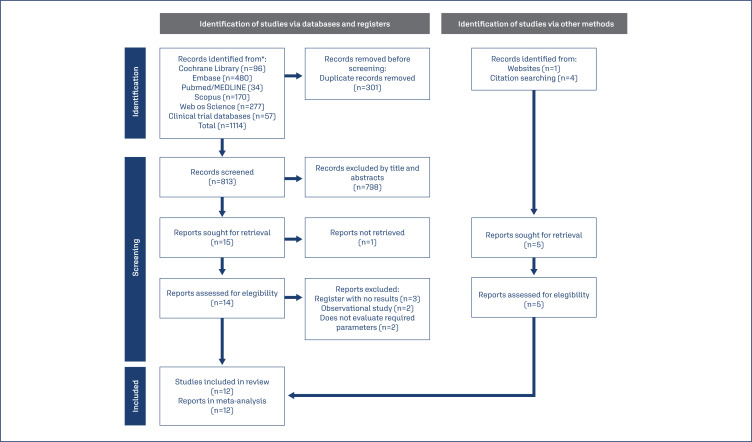
PRISMA flowchart

A total of 1114 studies were retrieved from the databases. After excluding 301 duplicates, 813 articles were left. Based on the inclusion selection criteria, authors read 15 articles for retrieval and assessed 14 full texts for eligibility. Together with the five studies identified from other sources, 12 RCT have been included in the final review.^(8,20,23–32)^ No additional study was selected after checking the reference lists from the eligible articles. Details of the study selection and review flowchart are presented in figure 1.

The 12 RCTs were published between 2017 and 2023 and involved patients from Iran,^(20)^ Australia,^(26)^ Belgium,^(32)^ Brazil,^(29–31)^ Greece,^(23,28)^ Italy,^(23)^ Thailand,^(25,27)^ and USA.^(8,24)^ All clinical trials were included in the qualitative synthesis and meta-analysis. The included RCTs had a total of 5147 participants who were randomized to receive laser therapy, estrogen, promestriene or sham-laser (placebo), with a mean age ranging from 57.6 to 63.1 years. The CO2-laser was used in 11 RCTs,^(8,20,23,24,26–32)^ whereas Erbium: YAG laser was used in only one.^(25)^ Among the included clinical trials, ten^(8,20,23,25–28,30–32)^ provided data on vaginal health index (VHI) and female sexual function index (FSFI), 8^(8,23–26,28,30,32)^ on dyspareunia and dysuria and only a few evaluated outcomes such as urinary incontinence,^(20,31)^ vaginal pH,^(26,31,32)^ and visual analog score (VAS).^(20,26,27,30,32)^ The characteristics of the included trials are presented in chart 2.

**Chart 2:** Characteristics of the studies included in the systematic review and meta-analysis

Author, year	Country	Study design	Intervention	Control/comparator	Participants, No.				Mean age (years)		Inclusion criteria	Therapeutic protocol	Follow-up duration	Outcomes measured
					Enrollment		Lost to follow up		Intervention group	Control group				
					Intervention	Control	Intervention	Control						
Cruz et al. (2018)^(30)^	Brazil	RCT	CO2-laser	Vaginal estriol	Randomized 45 Allocated 15 Analyzed 13	Randomized 45 Allocated 15 Analyzed 14	2	1	55.9 ± 5.2^a^	56.9 ± 6.0^a^	Aged 45–70 years who presented with amenorrhea for 24 months or longer and at least one moderate symptom of VVA.	2 laser therapies every 4 weeks	20 weeks	Primary outcomes include improvement of VHI and VVA symptoms (VAS). Secondary outcomes include improvement of sexual function (FSFI).
Girardelli et al. (2017)^(28)^	Greece	RCT	CO2-laser	Sham-laser	Randomized 55 Allocated 27 Analyzed 27	Randomized 55 Allocated 28 Analyzed 28	0	0	-	-	Women with postmenopausal VVA.	3 laser therapy every 1 month	4 months	FSFI, UDI-6, VAS for various genital symptoms (more specifically dyspareunia, dysuria, vaginal dryness, vaginal itching and vaginal burning sensation).
Ruanphoo and Bunyavejchevin (2020)^(27)^	Thailand	RCT	CO2-laser	Sham-laser	Randomized 63 Allocated 31 Analyzed 29	Randomized 63 Allocated 32 Analyzed 30	2	2	-	-	Postmenopausal women at fifty years of age or more with moderate to severe intensity of any symptoms of vaginal atrophy.	3 laser therapies every 4 weeks	12 weeks	VHI, VAS, and scores of ICIQ-VS questionnaire in terms of vaginal dryness, vaginal symptom, sexual matter and quality of life.
Politano et al. (2019)^(31)^	Brazil	RCT	CO2-laser	Promestriene IV 10mg Vaginal gel lubrificant	Randomized 72 Allocated 24 Analyzed 24	Promestriene group Randomized 72 Allocated 24 Analyzed 24 Lubrificant group Randomized 72 Allocated 24 Analyzed 24	2	Promestriene group 5 Lubrificant group 9	57.8 ± 5.0^a^	Promestriene group 57.2 ± 5.7^a^ Lubricant group 56.8 ± 5.3^a^	Aged 50 to 70 years; physiological amenorrhea for at least 12 months; symptoms of vaginal dryness with or without dyspareunia, vaginal burning, or pruritus; and no use of hormonal medications to treat vaginal symptoms in the prior 6 months.	3 laser therapies every 30 days	14 weeks	Vaginal maturation, VHI score, and sexual function (FSFI)
Singwongsa and Vallibhakara (2019)^(25)^	Thailand	RCT	Erbium: YAG laser	Sham-laser	Randomized 34 Allocated 17 Analyzed 17	Randomized 34 Allocated 17 Analyzed 17	-	-	-	-	-	3 laser therapies every 4 weeks	12 weeks	VAS, VHI, vaginal pH, VMI
Aguiar et al. (2020)^(29)^	Brazil	RCT	CO2-laser	Promestriene IV 10mg Vaginal lubrificant	Randomized 72 Allocated 24 Analyzed 24	Promestriene group Randomized 72 Allocated 24 Analyzed 24 Lubrificant group Randomized 72 Allocated 24 Analyzed 24	2	Promestriene group 5 Lubrificant group 7	57.8 ± 5.0^a^	Promestriene group 57.2 ± 5.3^a^ Lubrificant group 56.8 ± 5.3^a^	Women aged ≥ 50 years, who were amenorrhoeic for at least 1 year, with symptoms related to GSM, and who did not use hormonal therapy for at least last 6 months previously and any kind of medication for OAB (oral anti-muscarinics or oral β3-adrenoceptor agonists).	3 laser therapies every 30 days	14 weeks	Urinary symptoms (ICIQ-UI SF, and the ICIQ-OAB).
Li et al. (2021)^(26)^	Australia	RCT	CO2-laser	Sham-laser	Randomized 85 Allocated 43 Analyzed 38	Randomized 85 Allocated 42 Analyzed 40	5	2	55 ± 7^a^	58 ± 8^a^	Women aged 18 years and older who were fluent in English and had not previously received vaginal energy-based treatment for menopausal symptoms. Participants must have been amenorrheic for at least 12 months, either naturally or iatrogenically, and experiencing 1 or more of the following vaginal symptoms: dyspareunia, burning, itching, or dryness severe enough to prompt presentation to seek further treatment.	3 laser therapies every 1 month (minimum, 4 weeks; maximum, 8 weeks)	12 months	Severity of overall vaginal symptoms and individual symptoms of dyspareunia, dysuria, vaginal dryness, burning, and itching were assessed on a VAS; The VSQ is a 21-item validated instrument to measure vulvovaginal symptoms in postmenopausal women. This questionnaire assesses 4 domains (symptoms, emotions, life impact, and sexual-impact); Assessment of Quality of Life–6D scale13 and sexual satisfaction (Monash University Women's Health Program Female Sexuality Satisfaction Questionnaire; Vaginal Health Index Score (VHI- vaginal elasticity, vaginal fluid amount, pH, epithelial integrity, and hydration); vaginal skin biopsy (changes to collagen (reduced type I to type III fibril ratio, loss of trabecular disposition, absent fibrillogenesis), lamina propria (absence of activated fibroblasts, loss of mucopolysaccharides), epithelium (thinning of superficial layer; superficial keratinization; loss of rugae, elastic fibers, and subepithelial papillae; increase in subepithelial connective tissue), and decreased vascularization).
Paraiso et al. (2020)^(8)^	USA	RCT	CO2-laser	Vaginal estrogen cream	Randomized 69 Allocated 34 Analyzed 30	Randomized 69 Allocated 35 Analyzed 32	2	1	61 ± 8^a^	60 ± 7^a^	Menopausal women with absence of menstruation for at least 12 months and reported bothersome vaginal dryness of ≥7 cm on VAS.	3 laser therapies every 1 month	6 months	The primary outcome includes improvement of vaginal dryness (VAS). Secondary outcomes included VHI, VMI scores, quality of life (DIVA), sexual function (FSFI) and the urinary symptoms (UDI−6).
Salvatore et al. (2021)^(23)^	Greece and Italy	RCT	CO2-laser	Sham-laser	Randomized 60 Allocated 30 Analyzed 28	Randomized 60 Allocated 30 Analyzed 30	0	0	57.0 ± 6.9^a^	58.4 ± 6.0^a^	Postmenopausal women with GSM diagnosis according to the definition by the International Society for the Study of Women's Sexual Health and The North American Menopause Society, with no age limit. Dryness and dyspareunia related to GSM had to be the two most bothersome symptoms in all women.	3 laser therapies every 1 month	4 months	Changes in dryness and dyspareunia intensity; Changes in aspects of sexual functioning; Changes in itching; Burning, and dysuria intensity; Changes in UDI-6; Changes in dryness and dyspareunia incidence; Changes in sexual dysfunction incidence; Urinary incontinence; and Adverse Events.
Cruff and Khandwala (2021)^(24)^	USA	RCT	CO2-laser	Sham-laser	Randomized 30 Allocated 14 Analyzed 11	Randomized 30 Allocated 16 Analyzed 12	2	0	61 (54−66)^b^	59 (56−65)^b^	Menopausal women (or status-post bilateral oophorectomy) with dyspareunia or vaginal dryness rated as moderate-severe and who were desirous of sexual function, had vaginal atrophy based on a Gloria Bachmann VHI 28 score <15 and a vaginal pH > 5, prolapse less than Pelvic Organ Prolapse Quantification System (POP-Q)29 stage III, no pelvic reconstructive surgery within 6 months of treatment, ability to provide consent, and willing and able to attend all treatments and follow-up.	3 laser therapies every 6 weeks	6 months	Primary endpoint was improvement in GSM-related dyspareunia. Secondary objectives were to determine the impact of treatment on vaginal health, GSM symptoms, bothersome LUTS, sexual function, safety, and impact on quality-of-life.
Eftekhar et al. (2020)^(20)^	Iran	RCT	CO2-laser X RF	Sham-laser	CO2-laser group Randomized 246 Allocated 82 Analyzed 78 RF group Randomized 246 Allocated 82 Analyzed 80	Randomized 246 Allocated 82 Analyzed 79	CO2-laser 4 RF group 2	3	CO2-laser group 56.3 ± 7.2^a^ and RF group 57.7 ± 7.3^a^	54.8 ± 11.5^a^	Women with history of UI symptoms, sexual intercourse, sexual problems due to VVA, at least one year after the termination of menstruation, the presence of VVA symptoms, and sexual dysfunction.	CO2-laser group 3 laser therapies every 4 weeks RF group 3 RF sessions every 4 weeks	6 weeks	Urinary incontinence was evaluated using the ICIQ. In addition, pelvic organ prolapse/urinary incontinence sexual questionnaire (PISQ-12), VHI and VAS were used to determine sexual satisfaction.
Page et al. (2023)^(32)^	Belgium	RCT	CO2-laser	Sham-laser	Randomized 60 Allocated 30 Analyzed 28	Randomized 60 Allocated 30 Analyzed 29	2	1	57.40 ± 7.07^a^	56.20 ± 6.30^a^	Women with moderate to severe symptoms of GSM (i.e. vaginal dryness, vaginal itching, vaginal burning, dyspareunia and dysuria) shown by an Most Bothersome Symptom score of 2 or more	3 laser therapies every 4 weeks	3 months	Dyspareunia; Vaginal dryness; Dysuria; Vaginal burning; VAS score; Vaginal burning; Vaginal itching; FSFI score; ICIQ-OAB score; VHI score; Vaginal pH

a Mean ± Standard Deviation;

b Median (interquartile range). Randomized controlled trials (RCT), Vulvovaginal Atrophy (VVA), Visual Analogue Scale (VAS), Female Sexual Function Index (FSFI), Intravaginal (IV), Genitourinary Syndrome of Menopause (GSM), Vaginal Health Index (VHI), Vaginal Maturation Index (VMI), International Consultation on Incontinence Questionnaire Overactive Bladder (ICIQ), Vulvovaginal Symptom Questionnaire (VSQ), Radiofrequency (RF), Urge urinary incontinence (UUI), Urinary Distress Inventory, Short Form (UDI-6)

**Figure 2:**
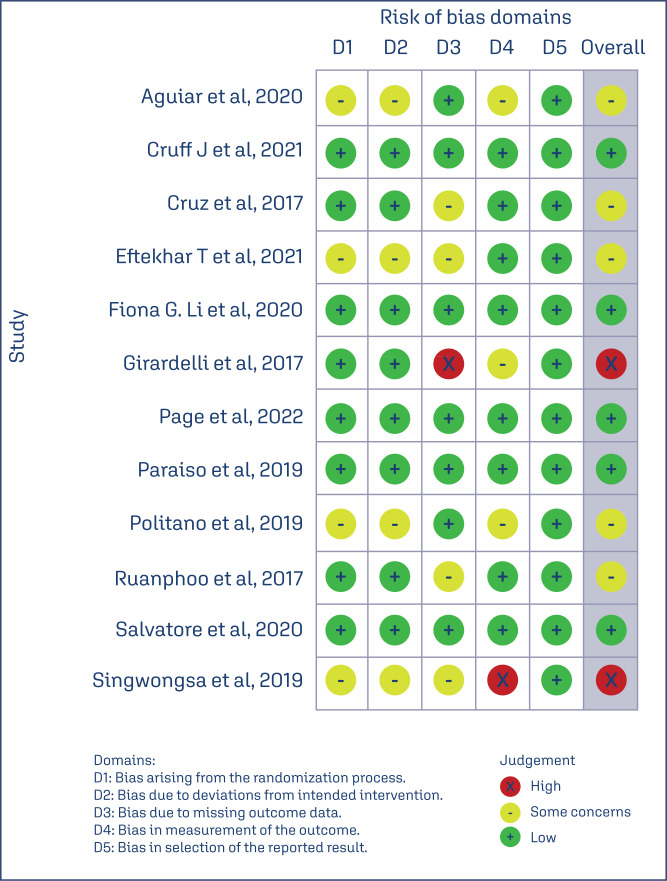
Risk of bias of included clinical trials

The risk of bias of each trial across 5 evaluated domains is shown in figure 2. Overall, five RCT had a low risk of reporting bias,^(8,23,24,26,32)^ five had some concerns^(20,27,29–31)^ and two had high risk of bias.^(25,28)^ Most of the trials presented some concerns and high risk due to the lack of clarity about the process of randomization and blinding.

**Figure 3:**
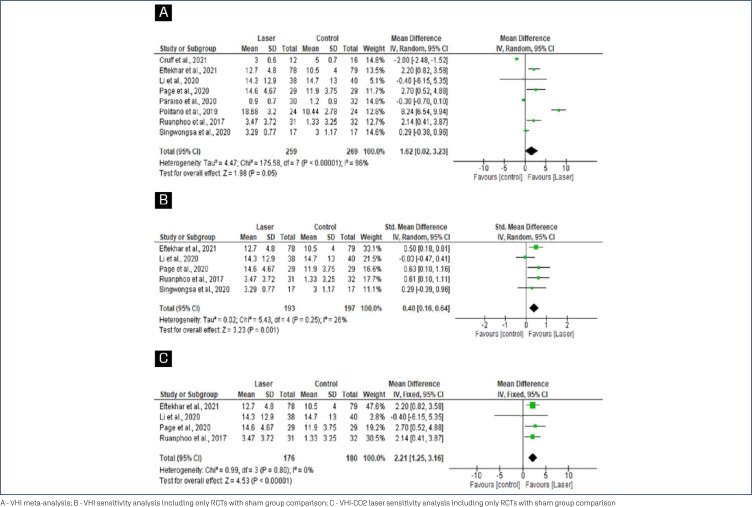
Forest plots of VHI

In the VHI meta-analysis (Figure 3), the mean differences (MD) were significantly greater among women randomized to receive laser therapy (MD=1.62; 95% CI=0.02 to 3.23). After sensitivity analysis, including only clinical trials that compared laser therapy with sham, an improvement was observed in the group that received laser with reduced heterogeneity (SMD=0.40; 95% CI=0.16 to 0.64). In addition, no heterogeneity was detected, especially when the analysis was performed with RCTs comparing CO_2-_ laser with sham-laser (MD=2.21; 95% CI=1.25 to 3.16), indicating that carbon dioxide laser therapy is effective in improving VHI.

A - VHI meta-analysis; B - VHI sensitivity analysis including only RCTs with sham group comparison; C - VHI-CO2 laser sensitivity analysis including only RCTs with sham group comparison

**Figure 4:**
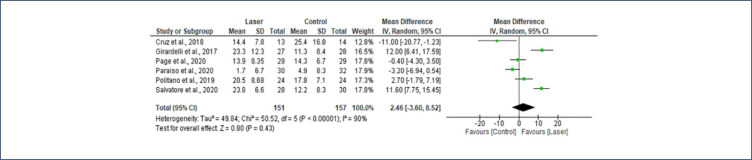
Female Sexual Function Index

When comparing the laser group with the control group, the MD in the pooled analysis for FSFI did not differ significantly between laser therapy and other therapies from baseline to the end of follow-up (MD= 2.46; 95% CI=−3.60 to 8.52), as shown in figure 4.

**Figure 5:**
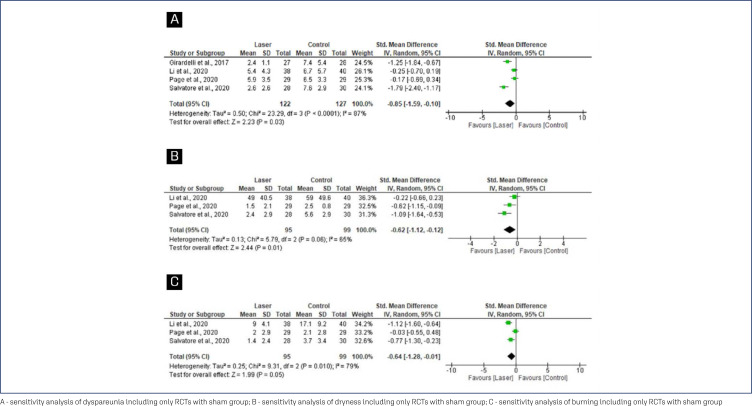
Forest plots of dyspareunia, dryness and burning

The SMD in the pooled analysis for dyspareunia, dryness and burning differ significantly between carbon dioxide laser therapy and sham-laser from baseline to the end of follow-up (SMD=−0.85; 95% CI=−1.59 to −0.10), (SMD=−0.62; 95% CI=−1.12 to −0.12) and (SMD= −0.64; 95% CI=−1.28 to −0.01), respectively (Figure 5).

A - sensitivity analysis of dyspareunia including only RCTs with sham group; B - sensitivity analysis of dryness including only RCTs with sham group; C - sensitivity analysis of burning including only RCTs with sham group

**Figure 6:**
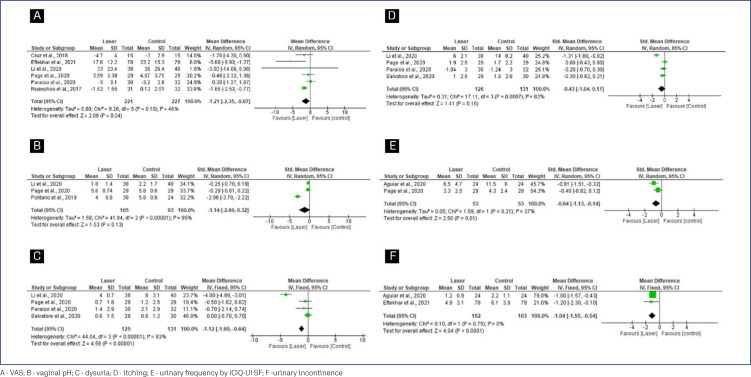
Forest plots of VAS, Vagina pH, Dysuria, Itching, Urinary frequency by ICIQ-UI SF and Urinary incontinence

The pooled analysis for the other outcomes (VAS, Vagina pH, Dysuria, Itching, Urinary frequency by ICIQ-UI SF and Urinary incontinence) (Figure 6).

A - VAS; B - vaginal pH; C - dysuria; D - itching; E - urinary frequency by ICIQ-UI SF; F -urinary incontinence

No serious adverse events were reported by the women treated with laser therapy. The most reported mild side effects were irritation, discomfort, and vaginal discharge. Egger's test did not reveal statistically significant publication bias for the RCTs that evaluated FSFI. For all other outcomes considered in the meta-analysis, the test indicated statistically significant publication bias. Chart 3 presents the certainty of evidence for each of the outcomes assessed in the RCTs. The primary outcome, VHI, as well as eight other outcomes, were classified as having low certainty of evidence according to GRADE guidelines. Vaginal Assessment Scale (VAS) and vaginal pH had very low certainty of evidence.

**Chart 3:** GRADE certainty of evidence

Outcomes	N° of participants (studies) Follow-up	Anticipated absolute effects	Certainty of the evidence (GRADE)
		Risk difference with [Laser therapy]	
Vaginal Assessment Scale (VAS)	448 (6 RCTs) 3 weeks-12 months	MD 1.21 lower (2.35 lower to 0.07 higher)	⨁⚪⚪⚪ Very low^a,b^
VHI	528 (8 RCTs) 4 weeks-12 months	SMD 0.4 SD higher (0.16 higher to 0.64 higher)	⨁⨁⚪⚪ Low
FSFI	308 (6 RCTs) 3 weeks-12 months	MD 2.46 higher (3.6 lower to 8.52 higher)	⨁⨁⚪⚪ Low^c^
Vaginal pH	198 (3 RCTs) 3 weeks-12 months	SMD 1.14 SD lower (2.6 lower to 0.32 higher)	⨁⚪⚪⚪ Very low^d,e,f,g^
Dyspareunia	249 (4 RCTs) 12 weeks-12 months	SMD 0.85 SD lower (1.59 lower to 0.1 lower)	⨁⨁⚪⚪ Low^h,i,j^
Dysuria	256 (4 RCTs) 3 weeks-12 months	MD 1.12 lower (1.6 lower to 0.64 lower)	⨁⨁⚪⚪ Low
Dryness	194 (3 RCTs) 12 weeks-12 months	SMD 0.62 SD lower (1.12 lower to 0.12 lower)	⨁⨁⚪⚪ Low
Burning	194 (3 RCTs) 12 weeks-12 months	SMD 0.64 SD lower (1.28 lower to 0.01 lower)	⨁⨁⚪⚪ Low
Itching	257 (4 RCTs) 3 weeks-12 months	SMD 0.43 SD lower (1.04 lower to 0.17 higher)	⨁⨁⚪⚪ Low
Urinary frequency (ICIQ-UI)	106 (2 RCTs) 3 weeks-12 months	SMD 0.64 SD lower (1.13 lower to 0.14 lower)	⨁⨁⚪⚪ Low
Urinary incontinence	205 (2 RCTs) 3 weeks-12 months	MD 1.04 lower (1.55 lower to 0.54 lower)	⨁⨁⚪⚪ Low

## Discussion

In this meta-analysis, conducted with 12 RCTs and more than 5000 participants, CO_2-_ laser therapy significantly improved VHI score and decreased dyspareunia, dryness and burning when compared to sham-laser. However, low certainty of evidence was observed for all these outcomes.

Initially, considering the pooled analysis of the 8 RCTs, in which there was a comparison of laser therapy with topical estrogen, promestriene and sham-laser, a slight improvement was found in the vaginal health index (VHI) of the participants treated with CO2-laser, or Er: YAG-laser. However, when analyzing only the results of clinical trials in which there was a comparison of CO2-laser with sham-laser, a significant improvement in VHI score was found in the group treated with CO2-laser without any heterogeneity. In a systematic review published in 2022,^(12)^ the authors concluded that further well-designed clinical trials with sham-laser control groups are needed in order to provide better evidence on the efficacy of CO2-laser therapy, which was confirmed in this pooled analysis with regard to a significant improvement in VHI scores in women treated with CO2-laser.

Mension et al.^(12)^ performed a systematic review in which they also concluded that the vaginal laser seems to improve scores on VHI. It should be noted that VHI evaluates 4 points upon the subjective criteria of the physician: elasticity, fluid volume, epithelial integrity, and moisture, and 1 point that can be objectively evaluated, which is the pH, with higher VHI scores indicating better vaginal health. This subjectivity probably influences the average scores found in clinical trials, especially in those in which the laser was compared with topical hormone vaginal therapy. This may explain the slight improvement observed when the results of the 8 RCTs were analyzed together.

The 2022 hormone therapy position statement of The North American Menopause Society (NAMS)^(2)^ reports that estrogen therapy (ET), specifically vaginal estrogen therapy (VET), is an effective treatment for GSM, with no evidence to suggest a difference in safety or efficacy between the various VET preparations. Thus, VET will likely increase VHI scores, justifying similar mean scores found in the groups randomized to CO2-laser and to VET. Therefore, it seems that when the RCTs that compared VET was removed from the meta-analysis and only the trials comparing CO2-laser to sham-laser were analyzed, VHI scores became significantly higher in laser-treated participants.

Another meta-analysis also showed a significant reduction in dyspareunia, dryness and burning in women treated with laser.^(33,34)^ Only a single study evaluated the certainty of the evidence using GRADE,^(34)^ in which the authors also classified the quality of the body of evidence as "low" or "very low". In our meta-analysis, the evidence was downgraded especially by inconsistency, uncertain or high risk of bias in most RCTs.

Some systematic reviews have concluded that CO2-laser has been associated with a significant improvement in Female Sexual Function Index (FSFI) score in comparison with that of the sham-laser group,^(12,13,33)^ which was not observed in this current meta-analysis. It seems that the inclusion of less RCTs in these previous studies and the fact that observational studies were included in only two of them might have resulted in the observed improvement in the FSFI, which was not confirmed when comparing the results of the 6 clinical trials included in this review that evaluated this outcome.

Furthermore, no significant differences were found between CO2-laser, sham-laser and VET from baseline to the end of follow-up regarding the VAS score, vaginal pH, itching and urinary frequency. Similar results were reported by Jang et al.,^(10)^ while Khamis et al.^(13)^ reported that CO2-laser was associated with a significant reduction in VAS and Urogenital Distress Inventory-6 scores when compared to those of the sham-laser group. The non-significant results found in this meta-analysis seems to be due to the small number of clinical trials in which these outcomes were evaluated. Another possible explanation may be the number of sections and the transient effect on these outcomes with the application of vaginal laser.

To the best of our knowledge, this is the RCTs meta-analysis that assessed the effectiveness of laser therapy in the management of GSM with the largest number of studies included. In addition, there was an assessment of the certainty of the evidence, as determined by GRADE, as well as the sensitivity analysis which was performed following Cochrane recommendations. However, it has some limitations, as most RCTs included had an uncertainty and high risk of bias, which makes us warn that the placebo effect cannot be ruled out. Finally, the different follow-up time in the included clinical trials, ranging from 4 to 24 weeks, and the lack of standardization in laser treatment might compromise the generalization of the results.

## Conclusion

Carbon dioxide laser (CO2-laser) increases VHI score and decreases dyspareunia, dryness and burning, especially when compared to sham-laser. However, the certainty of the evidence is low, preventing the recommendation of incorporating the laser therapy in the management of GSM until future studies demonstrate a significant improvement in the quality of the evidence.
